# LncTRPM2-AS inhibits TRIM21-mediated TRPM2 ubiquitination and prevents autophagy-induced apoptosis of macrophages in asthma

**DOI:** 10.1038/s41419-021-04437-6

**Published:** 2021-12-13

**Authors:** Xiaoping Li, Wenwen Wang, Yu Shao, Ji Zhou, Jiaqi Huang, Fei Xu, Xiu Gao, Mengyun Wu, Yongli Dong, Wenyan Wu, Jiamin Cai, Junyao Wang, Yunfei Ye, Zhengrong Chen, Chuangli Hao, Yi Yang, Jinping Zhang

**Affiliations:** 1 Institutes of Biology and Medical Sciences, Soochow University, Suzhou, People’s Republic of China; 2 Reproductive Medicine Center, The First Affiliated Hospital of Soochow University, Suzhou, People’s Republic of China; 3 Department of Respiratory Medicine, Children’s Hospital of Soochow University, Suzhou, People’s Republic of China

**Keywords:** Immune cell death, Respiratory tract diseases

## Abstract

Long non-coding RNAs (lncRNAs) play a crucial role in macrophage development but little is known about their role in asthma. Here, we investigated the role of lncRNA lncTRPM2-AS in asthma and found that lncTRPM2-AS participates in the promotion of macrophage inflammation. Downregulation of lncTRPM2-AS promoted apoptosis and inhibited proliferation and production of cytokines including IL-1β, IL-4, IL-6, IL-10, TNF-α, and TGF-β. RNA-immunoprecipitation and mass spectrometry indicated that the protein TRPM2 interacted with both lncTRPM2-AS and the E3 ubiquitin ligase TRIM21. LncTRPM2-AS silencing enhanced the interaction between TRIM21 and TRPM2, resulting in elevated levels of ubiquitin-related degradation of TRPM2. Mutation analysis indicated that TRPM2 K1218 is a key site for TRIM21-dependent ubiquitination. Downregulation of lncTRPM2-AS significantly decreased intracellular calcium levels by restraining TRPM2 protein expression, which in turn decreased ROS levels and increased autophagy to promote macrophage apoptosis and reduce cytokine production, together inhibiting macrophage inflammation. Taken together, our findings demonstrate that lncTRPM2-AS blocks the ubiquitination of TRPM2 via TRIM21 and inhibits autophagy-induced apoptosis which may contribute to macrophage inflammation in asthma.

## Introduction

Allergic asthma is one of the most common respiratory diseases, and there are an estimated 300 million asthmatics globally, with more than 30 million in China alone [1, 2]. Unfortunately, the treatment of asthma has not significantly improved in the past years [3]. Asthma development is generally ascribed to specific allergens such as air pollution and pollen [4]. Recent studies have indicated that after initial stimulation, allergens are recognized by pattern recognition receptors (PRRs) on the surface of respiratory epithelial cells and result in the recruitment of various immune cells including macrophages, eosinophils, and dendritic cells (DC) which can release cytokines such as IL-4, IL-5, IL-13, IL-33, IL-25, and granulocyte-macrophage colony-stimulating factor (GM-CSF). These mediators result in IgE production, accumulation and activation of eosinophils, airway abnormalities, and bronchial hyper-responsiveness, which ultimately lead to the occurrence of allergic asthma [5, 6]. To identify novel treatment strategies for asthma, it is of great significance to further explore its fundamental pathogenesis.

Macrophages are the most abundant immune cell in the lung and have both pro-inflammatory and anti-inflammatory functions. Macrophages have been shown to play a key role in the development of allergic asthma [7–9]. The two extremes of functional profiles of macrophages are described as classical activation (M1) and alternative activation (M2). M1 macrophages are stimulated by IFN-γ and lipopolysaccharide (LPS) and produce IL-1β and IL-6 in response to intracellular pathogens while M2 macrophages are induced by IL-4 and IL-13 [10]. Studies have shown that the numbers of alveolar macrophages are elevated in asthma and they showed increased levels of M2 polarization, with a particular increase of CD206^hi^MHC-II^hi^ M2-type macrophages [11]. It is reported that FoxO1 as a central effector molecule in the development of allergic inflammation suggests a new therapeutic approach to alleviate the suffering of TH2/M2 cell-related allergic diseases [12]. However, the specific molecular mechanisms of macrophage inflammation in the regulation of allergic asthma are only incompletely understood.

Long non-coding RNAs (lncRNAs) are non-coding transcripts longer than 200 nucleotides, which are thought to play major regulatory roles in many physiological and pathological pathways including cell proliferation, differentiation, and apoptosis, as well as innate and adaptive immunity, inflammation, tissue repair, and remodeling [13–15]. A disruption of lncRNA expression, elicited by alterations in the sequence, conformation, expression levels or patterns, or interaction with binding proteins, has previously been shown to be related to the occurrence of human diseases [16, 17], including tumors, ischemic reperfusion injury, and degenerative neurological diseases [18]. Although there is increasing evidence that lncRNAs participate in various key biological functions, their roles in the development of allergic asthma are largely unclear.

In this study, we assessed lncRNA expression profiles in PBMCs [18], which are frequently used for genomic analyses of asthmatic patients, and LPS-activated THP-1 cells using high-throughput sequencing, microarray, and real-time PCR. We found that lncTRPM2-AS was upregulated both in asthmatic patients and LPS-activated THP-1 cells. LncTRPM2-AS silencing significantly inhibited macrophage proliferation and cytokine production. Moreover, we assessed the interaction of lncTRPM2-AS with TRPM2 and TRIM21. Silencing of lncTRPM2-AS strengthened the interaction between TRIM21 and TRPM2 and resulted in increased levels of ubiquitin-dependent degradation of TRPM2. Further experiments presented in this article suggested that TRPM2 K1218 is the key site for TRIM21-dependent ubiquitination. Downregulation of lncTRPM2-AS significantly decreased intracellular calcium levels by inhibiting TRPM2 protein expression, in turn decreasing ROS and cytokine levels and promoting macrophage autophagy and apoptosis. Taken together, here we show for the first time that lncTRPM2-AS blocks TRIM21-dependent TRPM2 ubiquitination and thereby inhibits autophagy-induced apoptosis of macrophages in asthma.

## Results

### LncTRPM2-AS is highly expressed in asthmatic PBMCs and LPS-activated THP-1 cells

**Fig. 1:**
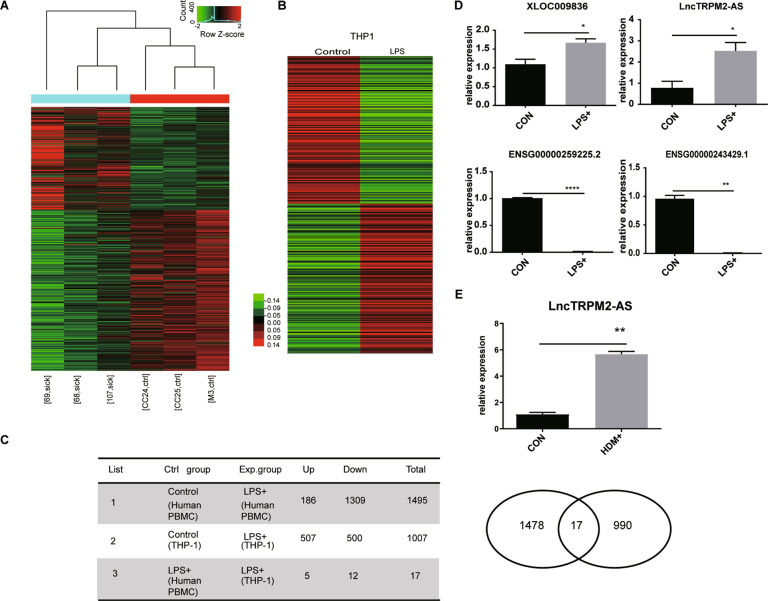
LncTRPM2-AS is highly expressed in PBMCs from asthmatic patients and LPS-stimulated THP-1 cells. **A** Expression profiles of lncRNAs in PBMCs from asthmatics and healthy controls. sick, asthmatic person PBMCs; ctrl, healthy person PBMCs. **B** Expression profiles of lncRNAs in LPS-stimulated THP-1 cells. **C** High-throughput sequencing data analysis and microarray analysis, differential expression analysis and cross-integration of data. **D** Four lncRNAs that were upregulated or downregulated both in asthmatic PBMCs and LPS-stimulated THP-1 cells were selected for RT-PCR analysis. **E** High expression of lncTRPM2-AS in HDM-stimulated THP-1 cells by RT-PCR. Bars, ± SD; *n* = 3, *P < 0.05; **P < 0.01; ****P < 0.0001.

To explore the role of lncRNAs in allergic asthma, we conducted high-throughput sequencing to analyze lncRNA expression profiles in peripheral blood mononuclear cells (PBMCs) from asthmatics and healthy controls. As expected, we found a significant difference in lncRNA expression profiles in PBMCs derived from asthmatic patients versus healthy controls (Fig. 1A), suggesting that certain lncRNAs may be associated with the development of asthma. Previous studies strongly indicated that endotoxins may participate in chronic airways diseases, in particular asthma, and LPS has previously been shown to induce cell activation and result in the expression of a number of lncRNAs [19]. Therefore, we also assessed the expression of lncRNAs in THP-1 monocytes stimulated with LPS or not via microarray. We identified hundreds of lncRNAs that were expressed in both LPS-activated cells and non-stimulated cells (Fig. 1B). We identified five lncRNAs which were upregulated both in PBMCs from asthmatics and LPS-stimulated THP-1 cells and twelve downregulated lncRNAs (Fig. 1C). From these lncRNAs, we selected four (two upregulated and two downregulated) which we further verified via RT-PCR. Consistent with the array data, RT-PCR confirmed that XLOC009836 and lncTRPM2-AS were upregulated while ENSG00000259225.2 and ENSG00000243429.1 were downregulated in LPS-treated cells (Fig. 1D). Amongst these, we found that lncTRPM2-AS expression was also significantly elevated in a house dust mite-induced cellular asthma model (Fig. 1E). Therefore, we decided to further investigate the role of lncTRPM2 in macrophage inflammation.

### LncTRPM2-AS inhibits macrophage apoptosis and promotes cytokine production

**Fig. 2:**
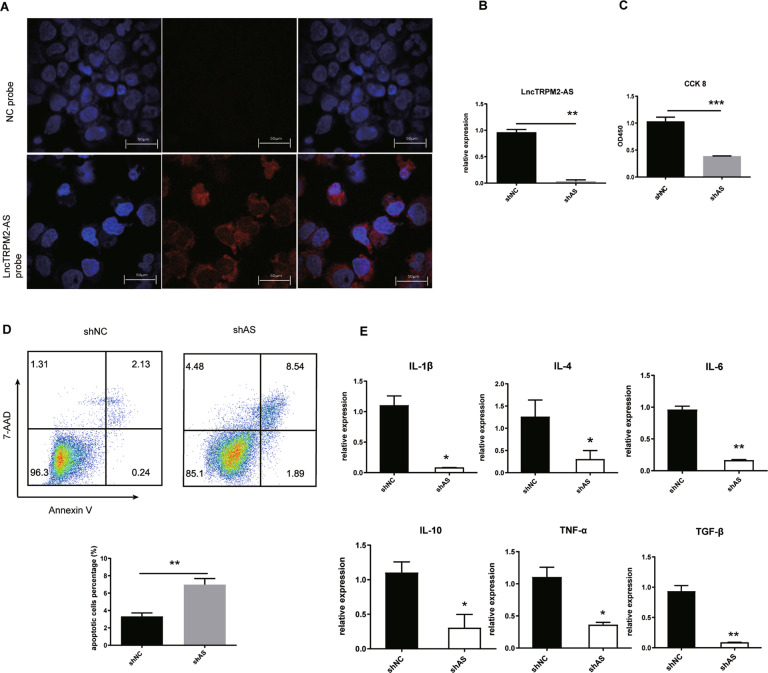
LncTRPM2-AS silencing promotes THP-1 cell apoptosis and inhibits cytokine production. **A** THP-1 cells were labeled with a lncTRPM2-AS probe (red) and negative control (NC) probe (red) using fluorescence in site hybridization (FISH), and counterstained with 4′,6-diamidino-2-phenylindole (DAPI) (nucleus staining, blue). **B** Confirmation of decreased lncTRPM2-AS levels following lncTRPM2-AS shRNA treatment. **C** Detection of cell proliferation in shNC or shlncTRPM2-AS (shAS) cells with the CCK-8 assay. **D** Detection of the apoptosis in shNC and shAS cells with the Annexin V/7-AAD assay. **E** Detection of the levels of IL-1β, IL-4, IL-6, IL-10, TNF-α, and TGF-β in shNC and shAS cells by RT-PCR. Bars, ± SD; *n* = 3, *P < 0.05; **P < 0.01; ***P < 0.001.

To investigate the functional role of lncTRPM2-AS in macrophages, we first explored the cellular localization of lncTRPM2-AS in THP-1 cells. Using fluorescence in situ hybridization (FISH), we found that lncTRPM2-AS was mainly located in the cytoplasm and cytomembrane (Fig. 2A), suggesting that it may exert its function predominantly in these cellular compartments. To further examine the functional role of lncTRPM2-AS in macrophages, we established a stable lncTRPM2-AS-silenced THP-1 cell line using shRNA. RT-PCR confirmed successful lncTRPM2-AS knockdown in this cell line, further referred to as shAS (Fig. 2B). We initially assessed cell proliferation and apoptosis and found a significant reduction in proliferation in the shAS cell line (Fig. 2C). Likewise, knockdown of lncTRPM2-AS resulted in elevated levels of apoptosis compared with control cells, as assessed by flow cytometry (Fig. 2D), indicating that lncTRPM2-AS plays an important role in preventing macrophage apoptosis. Moreover, we assessed the expression levels of cytokines such as IL-1β, IL-4, IL-6, IL-10, TNF-α, and TGF-β using RT-PCR which revealed that lncTRPM2-AS knockdown inhibited the production of these cytokines (Fig. 2E). These results propose that lncTRPM2-AS plays an important role in regulating macrophage apoptosis and inflammation.

### LncTRPM2-AS regulates TRPM2 protein stability

**Fig. 3:**
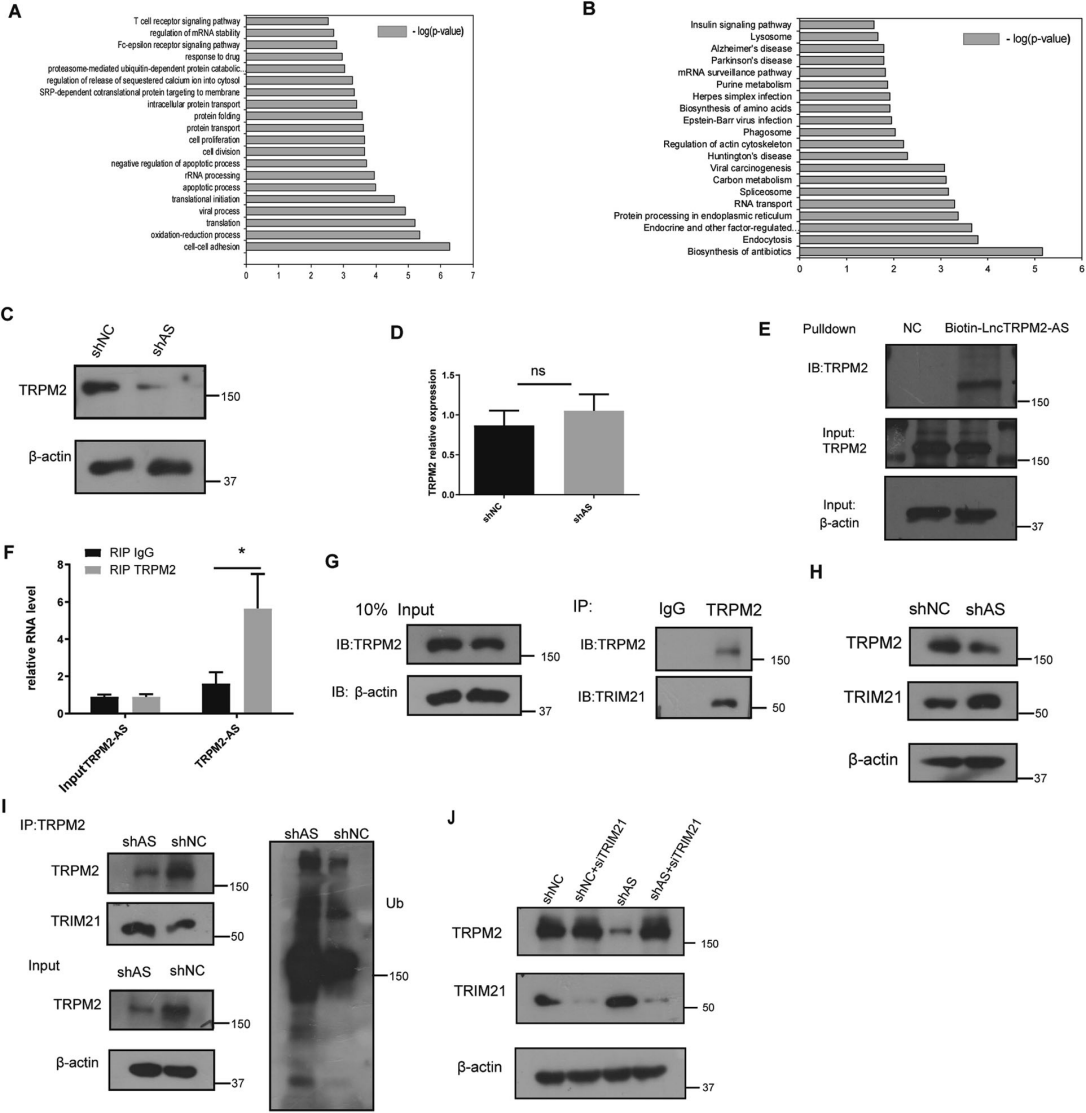
LncTRPM2-AS regulates TRPM2 protein expression. **A** GO analysis and **B** KEGG analysis showed the differentially expressed protein-coding genes in shNC and shlncTRPM2-AS cells. **C** TRPM2 protein levels were assessed by Western blot. **D** The experession of TRPM2 mRNA level in shAS cells was detected by qPCR. **E** The interaction between lncTRPM2-AS and TRPM2 was confirmed by RNA pull-down and Western blot. **F** RIP was performed using anti-TRPM2 and control IgG antibodies, followed by real-time PCR to examine the enrichment of lncTRPM2-AS and β-actin. β-actin served as the negative control. **G** The interaction between TRPM2 and TRIM21 was confirmed by co-immunoprecipitation (IP). **H** Detection of TRPM2 and TRIM21 protein in shNC and shAS cells. **I** TRPM2 antibodies were used for co-immunoprecipitation (IP) in shNC and shAS lysates, and TRIM21 and ubiquitin levels were detected. **J** Detection of TRPM2 and TRIM21 protein in shNC and shAS cells transfected with siTRIM21. Bars, ± SD; *n* = 3, ns not significant; *P < 0.05.

We next further investigated the molecular mechanisms by which lncTRPM2-AS may regulate macrophage inflammation. Mass spectrometry was conducted to evaluate the protein level expression change in shAS cells compared to shNC THP-1 cells. Moreover, GO and KEGG analysis revealed that lncTRPM2-AS knockdown resulted in changes in the expression of several proteins related to apoptosis, cell proliferation, protein poly-ubiquitination, and cytosolic calcium signaling (Fig. 3A, B). The first ten significantly reduced proteins were analyzed by mass spectrometry in shAS and shNC cells. As TRPM2 showed the highest fold change in molecules with a unique peptide detection greater than 10, we focused our further analyses on this protein (Supplementary Table 1). Western blotting confirmed that lncTRPM2-AS silencing significantly reduced TRPM2 protein levels (Fig. 3C), However, RT-PCR results showed no difference in TRPM2 mRNA level between shAS cells and shNC cells (Fig. 3D). This indicates that the regulation of the TRPM2 protein level may occur in post-translational modification. Interestingly, it has previously been reported that lncTRPM2-AS is transcribed from the antisense strand of TRPM2 gene [20], but there have not yet been any reports concerning the relationship between lncTRPM2-AS and TRPM2. We speculated that lncTRPM2-AS may bind to TRPM2 in macrophages and therefore performed an RNA pull-down assay using biotinylated lncTRPM2-AS to identify its binding partners. This demonstrated that lncTRPM2-AS was able to bind TRPM2 protein (Fig. 3E). Moreover, RNA-immunoprecipitation (RIP) also confirmed a specific binding between lncTRPM2-AS and TRPM2 (Fig. 3F). These results demonstrated that lncTRPM2-AS could bind to TRPM2 protein and might influence macrophage inflammation by regulating TRPM2 protein levels.

To further explore how lncTRPM2-AS regulates TRPM2 protein expression, we performed a pull-down experiment for TRPM2 in shNC and shAS cells to identify TRPM2-interacting proteins by mass spectrometry (MS). MS results indicated that TRIM21 was the most abundant E3 ubiquitin ligase in shAS among TRPM2-interacting proteins (Supplementary Table 2). This interaction was further confirmed by Co-IP in THP-1 cells (Fig. 3G). In addition, RIP experiments in THP1 cells showed that LnTRPM2-AS also interacted with TRIM21 (Supplementary Fig. 1). LncTRPM2-AS silencing increased TRIM21 protein levels while decreasing TRPM2 protein levels (Fig. 3H). Interestingly, Western blotting and Co-IP revealed that silencing of lncTRPM2-AS resulted in a stronger binding between TRIM21 and TRPM2 (Fig. 3I) and TRPM2 exhibited higher ubiquitination levels in lncTRPM2-AS silenced cells than in control cells (Fig. 3I). To further investigate the molecular mechanism of the regulation of TRPM2 by lncTRPM2-AS, TRIM21 siRNA were transfected into shNC or shAS cells, Western-blot showed that TRPM2 were increased in shAS cells after downregulation of TRIM21 (Fig. 3J). These results indicated that lncTRPM2-AS knockdown facilitates the interaction between TRIM21 and TRPM2, resulting in ubiquitin-dependent degradation of TRPM2.

### TRPM2 K1218 is the key site for TRIM21-dependent ubiquitination

**Fig. 4:**
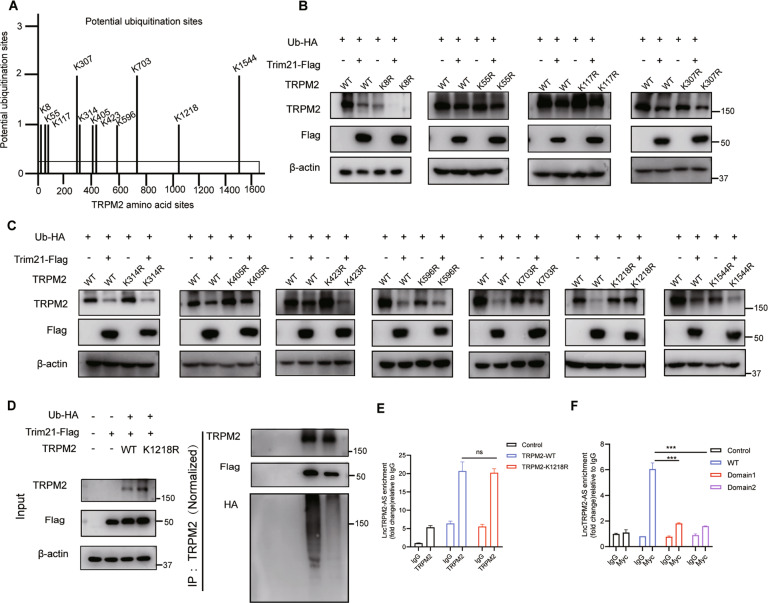
TRIM21 induces TRPM2 ubiquitination at Lys1218. **A** Potential ubiquitination sites of TRPM2 obtained from http://plmd.biocuckoo.org/. **B**, **C** Western blot analysis of TRPM2 in HEK293T cells co-transfected with Flag-Trim21, HA-Ub, and wild type or mutant TRPM2 (TRPM2 mutants: K8R, K55R, K117R, K307R, K314R, K405R, K423R, K596R, K703R, K1218R, and K1544R). **D** Immunoprecipitation analysis of ubiquitination of TRPM2 in HEK293T cells co-transfected with HA-Ub, Flag-TRIM21, and TRPM2 (WT or K1218R). **E** RIP experiments were used to detect the amount of lncTRPM2-AS after IP TRPM2. **F** RIP experiments were used to detect the amount of lncTRPM2-AS after IP Myc which represent different domains of TRPM2 as indicated. Bars, ± SD; *n* = 3, ns not significant; ***P < 0.001.

To identify the exact site of TRPM2 which was ubiquitinated by TRIM21, we searched a database of ubiquitination sites (http://plmd.biocuckoo.org) and found a total of 11 sites where TRPM2 may be ubiquitinated (Fig. 4A). We next constructed TRPM2-encoding plasmids with specific mutations at these 11 sites (K8R, K55R, K107R, K330R, K314R, K405R, K423R, K596R, K703R, K1218R, and K1544R) and confirmed the mutations by sequencing. To explore which sites contribute to TRIM21 ubiquitination, we co-transfected the above plasmids, Ub-HA, TRIM21-Flag, and TRPM2 into 293T cells and evaluated TRIM21-dependent ubiquitin degradation in the clones by Western blotting. The results indicated that only mutant K1218 TRPM2 could escape TRIM21-dependent degradation (Fig. 4B, C), which strongly indicated that the K1218 site of TRPM2 represents the key site for TRIM21-dependent ubiquitination. Further experiment suggested that mutation of TRPM2 K1218R abolished TRPM2 degradation mediated by TRIM21 (Fig. 4D). To clarify whether lncTRPM2-AS is through affecting the binding of TRIM21 to the ubiquitination site of TRPM2 to affect the ubiquitination degradation of TRPM2, we implemented two co-transfection experiments to detect the effect of lncTRPM2-AS on the ubiquitination area of TRPM2 and co-transfected lncTRPM2-AS, wild-type TRPM2 or mutant TRPM2 (K1218R) in 293T cells. RIP experiments were conducted to detect the amount of lncTRPM2-AS after IP of TRPM2. We found that there is no difference between wild-type and mutant TRPM2 for combination with lncTRPM2-AS (Fig. 4E). Therefore, we speculate that lncTRPM2-AS may bind to a certain structure of TRPM2 rather than an exact site to hinder the substrate ubiquitination by TRIM21. Based on this, we constructed a WT full-length plasmid of TRPM2 (0–4662 bp), Domain 1 (0–1092 bp), and Domain 2 (0–2937 bp), all of which are tagged with Myc and co-transfected with lncTRPM2-AS in 293T cells, The western blot demonstrated that the domains were constructed (Supplementary Fig. 2), The expression of lncTRPM2-AS was detected by Q-PCR after IP Myc, and the results showed that the binding of full-length TRPM2 to lncTRPM2-AS was significantly higher than that of domains 1 and 2 (Fig. 4F), indicating that lncTRPM2-AS mainly binds to the C-terminal part of TRPM2 including TRPM2 ubiquitinated site K1218R (nucleotide at position 3653). Therefore, lncTRPM2-AS spatially affects the ubiquitination of TRPM2 by TRIM21 via binding to the C-terminus of TRPM2 but not dependent on one amino acid site.

### LncTRPM2-AS increases calcium and ROS levels to decease autophagy-induced apoptosis

It has been reported that TRPM2, a member of the TRPM family, is involved in intracellular calcium signaling and oxidative stress [21]. Furmanik [22] stated that Ca2^+^ induces ROS production in VSMCs via Nox5. Consistent with this report, We used the TRPM2 channel activator cADPR to stimulate THP1 cells and then used flow cytometry to detect changes in cellular ROS. It was found that compared with the solvent group, the ROS level was indeed increased after the cells were treated with cADPR (Supplementary Fig. 3).

**Fig. 5:**
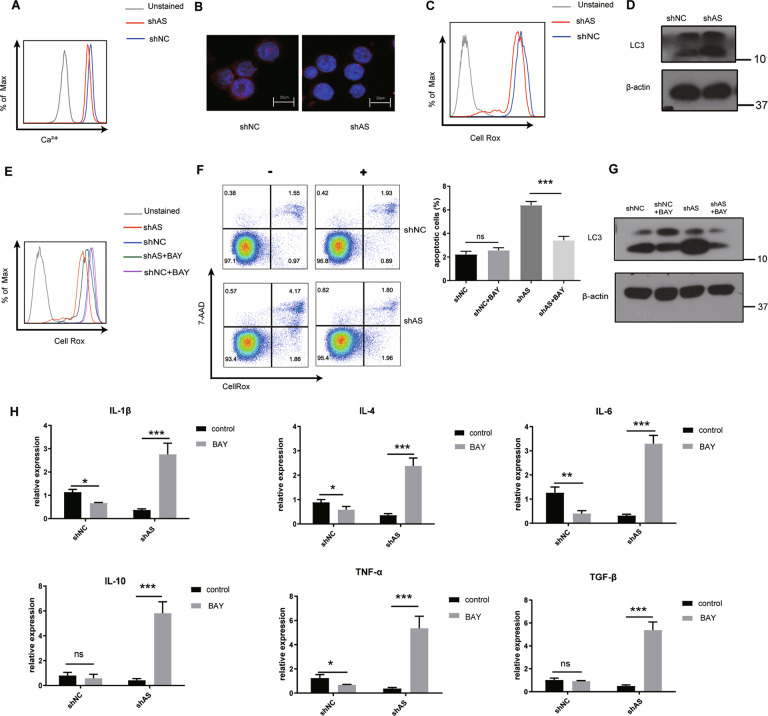
LncTRPM2-AS modulates macrophage apoptosis and cytokine production by regulating calcium levels. **A** Calcium and **C** ROS levels were detected by flow cytometry. **B** The density of calcium in a single shAS cell and shNC cell was detected by Confocal laser scanning microscopy. **D** Detection of LC3 protein in shNC and shAS cells by Western blotting. **E** ShNC and shAS cells were treated with a final concentration of 10 μM calcium activator BAY-K-8644 and the levels of ROS, **F** apoptosis, **G** LC3 protein, or **H** cytokines including IL-1β, IL-4, IL-6, IL-10, TNF-α, and TGF-β, were detected. Bars, ± SD; *n* = 3, ns not significant; *P < 0.05; ***P < 0.001.

cADPR activates the TRPM2 channel to promote the increase of calcium, which explains the increase of ROS. LncTRPM2-AS silencing decreased TRPM2 protein level. Thus, we assessed cellular calcium and ROS levels in control and lncTRPM2-AS-silenced cells using flow cytometry, which revealed that both calcium and ROS were decreased after silencing of lncTRPM2-AS (Fig. 5A, C). Consistent with the flow cytometry results, confocal laser scanning microscopy showed that the density of the calcium in a single shAS cell was significantly lower than that in shNC group (Fig. 5B). Cellular ROS are known to regulate autophagy by activating multiple kinase-dependent signaling pathways to initiate autophagosome formation or autophagy degradation [23]. Autophagy can induce apoptosis via the modulation of specific signaling pathways [24]. Hence, we further wanted to assess whether ROS levels may affect autophagy levels in lncTRPM2-AS-silenced cells. This revealed that autophagy levels were elevated after silencing of lncTRPM2-AS (Fig. 5D) and led us to hypothesize that lncTRPM2-AS knockdown may result in cellular apoptosis due to high levels of autophagy. Calcium is known to promote the formation of ROS-producing enzymes and free radicals [25, 26]. To assess whether the impact of lncTRPM2-AS in apoptosis and autophagy in macrophages depends on calcium and ROS levels, we applied the calcium activator to rescue cellular calcium levels in lncTRPM2-AS-silenced cells. cADPR, which is a specific agonist for TRPM2 C-terminal domain [27], was used to rescue the calcium flux in lncTRPM2-AS silenced cells. Flow cytometry analysis indicated that the cellular calcium of shAS cells treated with cADPR could not be recovered (Supplementary Fig. 4A). These remind us that the function of TRPM2 agonist cADPR depends on the expression level of TRPM2, this is consistent with the previous report [28]. Since TRPM2 is degraded in shAS cells, using the TRPM2 channel activator cADPR cannot rescue the missing calcium in the cells, then we used the voltage-gated calcium channel activator Bay-K-8644 instead of cADPR. We found that Bay-K-8644 can restore calcium in shAS cells (Supplementary Fig. 4B). To our surprise, treatment with Bay-K-8644 completely abrogated the impact of lncTRPM2-AS knockdown on ROS levels (Fig. 5E), suggesting that ROS levels were indeed regulated by intracellular calcium. Moreover, calcium activator treatment in lncTRPM2-AS-silenced cells also reversed the impact on apoptosis (Fig. 5F) and autophagy levels (Fig. 5G) as well as expression levels of certain cytokines, including IL-1β, IL-4, IL-6, IL-10, TNF-α, and TGF-β (Fig. 5H). These results indicated that lncTRPM2-AS silencing decreased cellular ROS and increased autophagy levels in THP-1 cells by modulating calcium levels, promoting apoptosis, reducing cytokine expression, and ultimately resulting in reduced levels of macrophage inflammation.

## Discussion

Among various cell types involved in allergic asthma, it has become clear that macrophages play an important role in the pathogenesis of the disease [29, 30]. During the phases of initiation and regression of inflammation, macrophages are activated and polarized. For instance, M2 macrophages in the bronchoalveolar lavage fluid of asthmatic patients highly express histamine receptor H1 (HRH1) and E-cadherin, secrete high levels of chemokines, and promote eosinophil infiltration into the lungs [31]. Moreover, increased numbers of M1 macrophages are also associated with asthma severity [32]. These findings suggested that macrophages may play a critical role in the pathogenesis of asthma. Exploring the molecular mechanisms of how macrophages contribute to asthma may elucidate novel targets for its treatment. As novel groups of non-coding transcripts, lncRNAs have been shown to participate in many biological pathways and inflammatory diseases via various mechanisms [15–17]; for example, lncRNAs are involved in cellular signal transduction or regulation of gene expression by interfering with mRNA splicing [18]. Numerous studies have since shown a functional role of lncRNA in various inflammatory diseases, however, their role in allergic asthma remained to be elucidated.

Several long noncoding RNAs (lncRNAs) have previously been reported to act as modulators of various aspects in the pathogenesis of asthma [33, 34]. Xia et al. [34] demonstrated that lncRNA BAZ2B, which is upregulated in asthmatic children and mainly expressed in monocytes, has the potential to promote the M2 polarization of macrophages by stabilizing BAZ2B precursor mRNA to enhance its cis-targeting BAZ2B. Han et al. [33] observed that lncRNA PTPRE-AS1 increased the expression of receptor-type tyrosine protein phosphatase ε (PTPRE) to suppress IL-4-induced M2 macrophages via the MAPK/ERK 1/2 pathway in vitro. Via RNA pull-down and ChIP assays, the authors demonstrated that lncRNA PTPRE-AS1 directly binds to WDR5 to modulate H3K4 trimethylation of the PTPRE promoter to repress M2 macrophage activation. Moreover, the expression of lncRNA PTPRE-AS1 and PTPRE have been shown to be reduced in PBMCs from asthmatic patients and expression levels of two are positively correlated, which indicated a potential role for lncRNA PTPRE-AS1 in the attenuation of M2 macrophage-mediated inflammation. The above two papers focused on the study of the role of lncRNAs in M2 macrophage-mediated inflammation in asthma. In the present study, we conducted high-throughput sequencing of patient samples combined with microarray analysis of lncRNAs in LPS-stimulated THP1 cells and identified a common upregulated lncRNA, lncTRPM2-AS. We demonstrated that lncTRPM2-AS predominantly acts in the cytoplasm and cytomembrane of macrophages and knockdown of this lncRNA significantly reduced the expression of cytokines related to both M1 and M2 macrophages (IL-1β, IL-4, IL-6, IL-10, TNF-α, and TGF-β). At the same time, lncTRPM2-AS silencing promoted apoptosis. There is a complex interplay between autophagy and apoptosis; the two can both be activated by a variety of stress stimuli and share multiple regulatory molecules [35, 36]. It has previously been reported that activation of autophagy inhibits airway fibrosis, which is one of the characteristics of asthma [37]. In our study, lncTRPM2-AS knockdown increased levels of autophagy of THP1 cells. To assess the reason for the increase in autophagy after lncTRPM2-AS knockdown, we used mass spectrometry to identify changes in protein levels. This revealed a decreased expression of TRPM2, which is involved in the release of calcium ions and furthermore participates in pathological processes such as oxidative stress and cell death. In accordance with this, we found reduced calcium and ROS levels after silencing of lncTRPM2-AS. Conversely, treatment with the calcium ion agonist Bay-K-8644 rescued ROS levels in lncTRPM2-AS-silenced cells. Studies have previously suggested that asthma is a chronic inflammatory disease of the respiratory tract, which is caused by cellular mechanisms that produce increased levels of reactive oxygen species (ROS) [38]. Our experiments confirmed that knockdown of lncTRPM2-AS decreased ROS levels while increasing autophagy and ultimately leading to apoptosis; our data suggest that these effects were initiated by modulation of intracellular calcium levels via TRPM2.

Previous papers have shown that the E3 ligases Cbl-b, ITCH, and TRIM18 are associated with inflammation in asthma [39]. In our study, immunoprecipitation-mass spectrometry (IP-MS) in lncTRPM2-AS-silenced cells revealed an increased binding and subsequent ubiquitination of TRPM2 by ubiquitin ligase TRIM21. Some articles have used quantitative proteomics [40], IHCKSAAP_UBsite [41], quantification of 10,000 ubiquitination sites in a single proteomics experiment, and quantitative investigation of ubiquitination sites in vivo [42, 43] to predict potential ubiquitination sites in TRPM2 but few studies have reported the biological function of TRPM2 after ubiquitination. The ubiquitin-proteasome system is the major pathway for protein degradation, a fundamentally crucial process in eukaryotes [44, 45]. Using IP-MS, we found that the ubiquitin ligase TRIM21 interacted with TRPM2. Moreover, lncTRPM2-AS knockdown enhanced binding between TRIM21 and TRPM2, thereby resulting in increased levels of ubiquitination and degradation of TRPM2. After transfecting control or lncTRPM2-AS-silenced cells with siTRIM21, TRPM2 protein levels were essentially restored to normal levels in lncTRPM2-AS silencing cells while there was no change in control cells.

It has been reported that TRPM2 is associated with cellular calcium channel and acts as a sensor of cellular ROS, which participates in many physiological and pathological processes [46, 47]. Here we found that calcium and ROS levels were both decreased in lncTRPM2-AS-silenced cells compared to controls. ROS can induce mTORC1 expression via inactivation and oxidation of PTEN and TSC1/2, thereby negatively regulating autophagy [48]. Interestingly, we found that knockdown of lncTRPM2-AS enhanced cellular autophagy levels. As autophagy is often associated with apoptosis, we found that apoptosis was induced by increased levels of autophagy in lncTRPM2-AS-silenced cells. Intracellular calcium levels are closely related to ROS production, activating ROS-producing enzymes and promoting the formation of free radicals [49]. Thus, we speculated that lncTRPM2-AS may regulate cellular ROS levels, autophagy, and apoptosis by influencing cellular calcium levels. By treating lncTRPM2-AS-silenced cells with a calcium activator (Bay-K-8644), we found that effects of lncTRPM2-AS silencing on ROS levels, autophagy, apoptosis, and cytokine expression were reversed. Collectively, our data suggest that lncTRPM2-AS regulates macrophage apoptosis and cytokine production by modulating intracellular calcium levels.

In summary, lncTRPM2-AS blocks TRIM21-dependent ubiquitination of TRPM2 and inhibits autophagy-induced apoptosis of macrophages in asthma. LncTRPM2-AS may be used as a diagnostic indicator and molecular target for asthma treatment, although extensive preclinical and clinical research will be required prior to the use of molecular targets in the clinic.

## Methods

### High-throughput sequencing and microarray analysis of lncRNAs

PBMCs derived from asthmatic patients and healthy controls were extracted and re-suspended in Trizol plus reagent. Total RNA and lncRNA sequencing was performed by Cloud Seq Biotech in Shanghai, China. High-throughput sequencing data has been deposited in NCBI Gene Expression Omnibus (GEO) under the accession number GSE174325. LPS-treated and untreated THP-1 cells were collected in Trizol plus reagent. Microarray analysis of total RNA and lncRNA was performed by Boao Biotech in Guangzhou, China. Microarray data has been deposited in NCBI Gene Expression Omnibus (GEO) under the accession number GSE174197.

### Plasmids

Full-length human TRPM2 (Transient receptor potential cation channel subfamily M member 2) DNA was purchased from Addgene and eleven mutant constructs (K8R, K55R, K117R, K307R, K314R, K405R, K423R, K596R, K703R, K1218R, and K1544R) were generated using specific primers (shown in Supplementary Table 3), the PrimeSTAR® GXL DNA Polymerase (TAKARA) and FastDigest DpnI (Thermo Fisher Scientific) according to the manufacturer′s instructions. A TRIM21-Flag plasmid was kindly provided by Dr. Wei Xu (Soochow University, Suzhou, China). To construct the shlncTRPM2-AS plasmid, the primers (F: AACGCGGTTACGAGGGCAAATATTCAAGAGATATTTGCCCTCGTAACCGCTTTTTTC, R: TCGAGAAAAAAGCGGTTACGAGGGCAAATATCTCTTGAATATTTGCCCTCGTAACCGCGTT) were inserted into vector pLL3.7, and the presence of the plasmid was confirmed by sequencing and restriction enzyme digest using Hpa I and Xho I.

### Cell culture, lentiviral infection, and transfection

Human THP-1 cells were purchased from ATCC and cultured in RPMI-1640 medium (HyClone, Logan, Utah) with 10% FBS (Gibco, Hong Kong) containing 1% penicillin–streptomycin solution and 0.05 mM β-Mer (Invitrogen, Camarillo, CA, USA) at 37 °C in a humidified 5% CO_2_ atmosphere. THP-1 cells were infected with the LV5 (EF-1aF/GFP&Puro) lentiviral vector carrying the target gene sequence or a scrambled shRNA purchased from GenePharma. Inc (Shanghai, China) according to the manufacturer’s protocol. To select cells expressing the plasmids, cells were selected with puromycin (Solarbio, Beijing, China) or sorted using a BD FACS Arial III. The human siTRIM21 sequence (UGGCAUGGAGGCACCUGAAGGUGG) was ordered from GenePharma. Inc (Shanghai, China). The siRNAs were transfected at 20 nM final concentration using the FECTTM CP Reagent (Ribo, Guangzhou, China) and analyzed 48 h after transfection according to the manufacturer’s instruction.

### Real-time PCR

Total RNA of cells was extracted using RNAiso Plus reagent (Takara Biotechnology Co. LTD) and reverse transcribed into cDNA using a PrimeScript^TM^ RT reagent kit (Takara Biotechnology Co. LTD) according to the manufacturer’s protocol. Quantitative real-time PCR was performed in triplicate by using FastStart Universal SYBR Green Master (Roche, Shanghai, China) on an Eppendorf Real-Time Detection System (Eppendorf, Shanghai, China). The PCR program was 95 °C for 10 min, (95 °C for 15 s, and 60 °C for 60 s, this part of the program was repeated for 40 amount of cycles) and included a melting curve. The primer pairs used for LncTRPM2-AS, TRPM2, IL-1β, IL-4, IL-6, IL-10, TNF-α, TGF-β, and β-actin are listed in Supplementary Table 4.

### Apoptosis assay

For the apoptosis assay, single cell suspensions were stained with APC-Annexin V (Biolegend, San Diego, CA, USA) and 7-Amino-Actinomycin (7-AAD) (BD Pharmingen, San Jose, CA, USA) in an Annexin V binding buffer according to the manufacturer’s instruction. Cells were analyzed using a BD FACS CantoII. FACS data was quantified using the FlowJo software (Tree Star, Ashland, OR, USA).

### CCK-8 assay

For the CCK-8 assay, cells were incubated in 96-well plates and 10 μL CCK-8 reagent (Sangon Biotech, Shanghai, China) was added into each well according to the manufacturer’s protocol. The absorbance wavelength 450 nm was detected using a Synergy 2 microplate reader (BioTek, Winooski, VT, USA).

### RNA FISH

For fluorescence in situ hybridization (FISH), lncRNA-fluorescence conjugated probes were designed by Ribo Biotech in Guangzhou, China. The RNA FISH experiment was performed using the Fluorescent In Situ Hybridization Kit (Ribo). Samples were counterstained with DAPI (4,6-diamidino-2-phenylindole; Sigma-Aldrich) and images were acquired using a confocal microscope.

### RNA pull-down assay

Biotin-labeled lncTRPM2-AS was constructed in vitro using a Biotin RNA Labeling Mix and T7 RNA polymerase (Ribo) according to the manufacturer’s instructions. Whole cell lysates (50 μg per sample) were incubated with 1 μg of biotin-labeled RNA overnight at 4 °C and complexes were purified with Streptavidin agarose beads (Invitrogen) for 4 h at 4 °C. The expected proteins in the complexes were detected using Western blot analysis while expected RNAs in the complexes were detected by RT-PCR analysis.

### RIP assay

RNA-binding protein immunoprecipitation (RIP) was performed using the RIP kit (MBL) according to the manufacturer’s protocol. The relative cell lysates were harvested using the RIP lysis buffer and incubated with control IgG or TRPM2 antibody (Abcam, ab11168) overnight at 4 °C. The interaction complexes were precipitated using Protein G beads (Roche) and RNAs were detected by RT-PCR.

### Cellular calcium detection

Cellular calcium levels were assessed using Calcium Orange^TM^ (Invitrogen, Camarillo, CA, USA). Cells were stained with 10 μM Calcium Orange^TM^ for 1 h at room temperature and washed with PBS to remove excess probe. The mean fluorescence was determined using the BD FACS Arial III. FACS data was analyzed using the FlowJo software (Tree Star, Ashland, OR, USA). The calcium density is either observed under confocal laser scanning microscope (CLSM). After cells were stained with 10 μM Calcium Orange^TM^, Cells were then washed with PBS and fixed with 4% paraformaldehyde in PBS for 20 min, permeabilized with 0.2% Triton X-100 and blocked with 3% bovine serum albumin. Nuclei were counterstained with DAPI (4, 6-diamidino-2-phenylindole; Sigma-Aldrich).

### ROS detection

Cellular ROS levels were detected using CellROX® Deep Red reagent (Invitrogen). Briefly, cells were treated with a final concentration of 5 μM CellROX® Deep Red reagent for 30 min at 37 °C and cells were subsequently washed three times with PBS. The mean fluorescence was determined using the BD FACS Arial III. FACS data were analyzed using the FlowJo software (Tree Star, Ashland, OR, USA).

### Western blot analysis

Cell lysates were obtained from cultured cells using RIPA lysis buffer (Beyotime, P0013C) containing protease inhibitors PMSF (Sigma) for 20 min on ice. The BCA Protein assay kit (Thermo Scientific) was used to quantify protein concentration in the samples. Prior to loading, 5× SDS loading buffer was added to protein lysates and the samples were boiled for 10 min. Samples were separated using SDS-PAGE gel and transferred to polyvinylidene fluoride (PVDF) membranes. Membranes were blocked in 5% milk and incubated with the following antibodies overnight at 4 °C: anti-β-Actin (1:1000, mouse polyclonal, Cell Signaling Technology, 3700), anti-Ubiquitin (1:1000, rabbit polyclonal, Cell Signaling Technology, 43124), anti-TRPM2 (1:1000, rabbit polyclonal, Abcam, ab96785), anti-TRIM21 (1:200, mouse polyclonal, Santa Cruz, sc-25351), and anti-LC3 (1:1000, rabbit polyclonal, Cell Signaling Technology, 12741). Blots were then incubated in HRP-conjugated secondary antibodies, treated with enhanced chemiluminescence (ECL) reagents (Bio-Rad), and protein bands were observed by exposure to X-ray films (Kodak).

### Ubiquitination site detection

TRPM2 contains 85 lysine residues. Eleven of these (Lys8, Lys55, Lys117, Lys307, Lys314, Lys405, Lys423, Lys596, Lys703, Lys1218, and Lys1544) were predicted to be potential ubiquitination sites by the protein lysine modification database (http://plmd.biocuckoo.org/). To identify TRIM21-dependent ubiquitination sites of TRPM2, we replaced each of the eleven TRPM2 lysine residues noted above with arginine. HA-Ub, wild type, and mutant TRPM2 were transfected into 293T cells together with or without TRIM21-Flag. After 48 h, ubiquitination sites were assessed by Western blotting.

### Statistical analyses

GraphPad Prism 9.0 was used for statistical analysis. Data are expressed as mean ± SD. Differences between two groups or more than two groups were performed by Student’s *t*-test or ANOVA, respectively. *P* values less than 0.05 were considered statistically significant.

## Supplementary information

Supplemental materials

Supplementary Figure 1

Supplementary Figure 2

Supplementary Figure 3

Supplementary Figure 4

Checklist

## Data Availability

The data used and/or analyzed during the current study are available from the corresponding author on reasonable request.
