# Supplementary material for: LncTRPM2-AS inhibits TRIM21-mediated TRPM2 ubiquitination and prevents autophagy-induced apoptosis of macrophages in asthma
Source: Cell Death Dis. 2021 Dec 13;12(12):1153. doi: 10.1038/s41419-021-04437-6 (PMC8668916; doi:10.1038/s41419-021-04437-6)
Supplement: Supplementary file 1 — Supplemental materials [file 41419_2021_4437_MOESM1_ESM.doc]

**Supplementary methods**

**Cell treatment**

THP-1 , shNC or shAS cells were treated with 100 µM cADPR (MCE,HY-N7395) for 10 min or 10 µM Bay-K-8644 (MCE,HY-10588) for 24h. Then cells were collected for FACS, Real-time PCR and Western blot analysis.

**Construction of different domains of TRPM2**

WT full-length plasmid of TRPM2 (0-4662bp), domain1 (0-1092bp), and domain 2 (0-2937bp), all of which are tagged with Myc constructed by Youbio (Youbio Biological Technology,China).

**RIP assay**

RNA-binding protein immunoprecipitation (RIP) was performed using the RIP kit (MBL) according to the manufacturer's protocol. The relative cell lysates were harvested using the RIP lysis buffer and incubated with control IgG or Myc antibody (CST, 9B11) overnight at 4℃. The interaction complexes were precipitated using Protein G beads (Roche) and RNAs were detected by RT-PCR.

**Mass spectrometry**

ShNC and shAS cells were harvested, then sonicated three times on ice using a high intensity ultrasonic processor (Scientz) in lysis buffer containing 150 mM NaCl, 10 mM HEPES, 8M urea and protease inhibitors mixtures (Sigma). The remaining debris was removed by centrifugation at 12,000 g at 4 °C for 10 min. Finally, the supernatant was collected and the protein concentration was determined with a BCA kit according to the manufacturer’s instructions. For digestion, the protein solution was reduced with 5 mM dithiothreitol for 30 min at 56 °C and alkylated with 11 mM iodoacetamide for 15 min at room temperature in darkness. Finally, trypsin (Thermo Fisher) was added at 1:50 trypsin-to-protein mass ratio overnight. The tryptic peptides were purified using C18 Zip Tip. Then the peptides were analyzed by an Orbitrap Elite hybrid mass spectrometer (Thermo Fisher) coupled with a Dionex LC. The resulting MS data were processed using Proteome Discoverer 1.2. Tandem mass spectra were searched against UniProt database concatenated with reverse decoy database. The mass tolerance for fragment ions was set as 0.02 Da. Systematic bioinformatics analysis of proteins including protein annotation, function classification, function enrichment were conducted. For immunoprecipitation, shNC and shAS cells were harvested by Nonidet P-40 lysis buffer containing 150 mM NaCl, 20 mM Tris-HCl (PH7.4), 1% NP-40, 0.5 mM EDTA, PMSF (50 μg/mL) and protease inhibitors mixtures . M2 affinity gel (A2220; Sigma-Aldrich) was used to pull down TRPM2 antibody from the whole cell lysates. SDS-PAGE gels were stained with the Silver Staining kits (Beyotime, P0017S). The gel bands from control and experimental samples were carefully excised, and then were digested with trypsin. The subsequent steps are the same as the above steps of whole protein mass spectrometry.

**Supplementary Table 1** The first 10 significantly reduced proteins with a unique peptide detection greater than 5 were analyzed by mass spectrometry in shAS and shNC cells.

| Accession | Description | Unique Peptides | MW [kDa] | Ratio: (shAS) / (shNC) |
| --- | --- | --- | --- | --- |
| Q99541 | PLIN2 | 5 | 48 | 0.243 |
| Q32MZ4 | LRRFIP1 | 5 | 89.2 | 0.285 |
| O94759 | TRPM2 | 17 | 171.2 | 0.508 |
| Q9Y3L3 | SH3BP1 | 5 | 75.7 | 0.523 |
| P31146 | CORO1A | 10 | 51 | 0.552 |
| Q16555 | DPYSL2 | 17 | 62.3 | 0.573 |
| Q05639 | EEF1A2 | 5 | 50.4 | 0.578 |
| Q16822 | PCK2 | 12 | 70.7 | 0.582 |
| P35527 | KRT9 | 8 | 62 | 0.584 |
| P08243 | ASNS | 11 | 64.3 | 0.587 |

**Supplementary Table 2** E3 ubiquitin ligases were screened from IP mass spectrometry data in shAS cells.

| Accession | Description | Unique Peptides | MW [kDa] |
| --- | --- | --- | --- |
| P49792 | RANBP2 | 1 | 358 |
| O60858 | TRIM13 | 1 | 47 |
| Q14258 | TRIM25 | 4 | 70.9 |
| P19474 | TRIM21 | 30 | 54.1 |
| P61289 | PSME3 | 2 | 29.5 |
| O75592 | MYCBP2 | 16 | 513.3 |
| Q7Z6Z7 | HUWE1 | 3 | 481.6 |
| Q9NS91 | RAD18 | 3 | 56.2 |

**Supplementary Table 3** Sequences of primers used in point mutation.

| **Number** | **Sequence (5’-3’)** | **Purpose** |
| --- | --- | --- |
| P1 | CCGAGCCAGCTCTCCTCAGGGCTGAG | Forward primer for K8R |
|  | CTCAGCCCTGAGGAGAGCTGGCTCGG | Reverse primer for K8R |
| P2 | GGCTTTCTTGCCTGTCATTGTTGCCGAAGGGGC | Forward primer for K55R |
|  | GCCCCTTCGGCAACAATGACAGGCAAGAAAGCC | Reverse primer for K55R |
| P3 | CCTGGACATGTCTCTTTGGGTCCCACTGTGTGC | Forward primer for K117R |
|  | GCACACAGTGGGACCCAAAGAGACATGTCCAGG | Reverse primer for K117R |
| P4 | TGCTCCGATATGAACCTCTCCAGCCTGGTCC | Forward primer for K307R |
|  | GGACCAGGCTGGAGAGTTCATATCGGAGCA | Reverse primer for K307R |
| P5 | CACCTCCTCTTTCCCTGGTCTGCTCCGATATGA | Forward primer for K314R |
|  | TCATATCGGAGCAGACCAGGGAAAGAGGAGGTG | Reverse primer for K314R |
| P6 | GACGATATCTTGGATCCTTTTGGTCCACTCGACAATC | Forward primer for K405R |
|  | GATTGTCGAGTGGACCAAAAGGATCCAAGATATCGTC | Reverse primer for K405R |
| P7 | CTGCTGACCATCCCTGCCTTCCCGGAAGAC | Forward primer for K423R |
|  | GTCTTCCGGGAAGGCAGGGATGGTCAGCAG | Reverse primer for K423R |
| P8 | GCACGTTGAGCCTGACGTGGGGAACGG | Forward primer for K596R |
|  | CCGTTCCCCACGTCAGGCTCAACGTGC | Reverse primer for K596R |
| P9 | CGGGTGAGCAGTCTCTGGGCTCTCTCTTCG | Forward primer for K703R |
|  | CGAAGAGAGAGCCCAGAGACTGCTCACCCG | Reverse primer for K703R |
| P10 | GGAGCCCGACCTCCTCAGTGGGTCCAG | Forward primer for K1218R |
|  | CTGGACCCACTGAGGAGGTCGGGCTCC | Reverse primer for K1218R |
| P11 | GCGGCTGCCCTCTGGAGGAGGGTCTTG | Forward primer for K1544R |
|  | CAAGACCCTCCTCCAGAGGGCAGCCGC | Reverse primer for K1544R |

**Supplementary Table 4** Sequences of primers used in qRT-PCR.

| **Name** | **Sequence (5’-3’)** |
| --- | --- |
| Human β-actin | F: GGGCATGGAGTCCTGTGGCA |
|  | R: GGGTGCCAGGGCAGTGATCTC |
| Human TRPM2-AS | F: CGTGACCAGGTTCAGACACA |
|  | R: TGGGCAGTTTGGTTCTGGTT |
| Human TRPM2 | F: AAGCCTCAGTTCGTGGATTC |
|  | R: TGAAGACGATGTCGCCAAAG |
| Human IL-1β | F：AGCTACGAATCTCCGACCAC |
|  | R: CGTTATCCCATGTGTCGAAGAA |
| Human IL-4 | F: TGCCTCACATTGTCACTGC |
|  | R: GCACATGCTAGCAGGAAGAAC |
| Human IL-6 | F: ACTCACCTCTTCAGAACGAATTG |
|  | R: CCATCTTTGGAAGGTTCAGGTTG |
| Human IL-10 | F: AAGACCCAGACATCAAGGCG |
|  | R: AATCGATGACAGCGCCGTAG |
| Human TNF-α | F: CTCTTCTGCCTGCTGCACTTTG |
|  | R: ATGGGCTACAGGCTTGTCACTC |
| Human TGF-β | F: CTAATGGTGGAAACCCACAACG |
|  | R: TATCGCCAGGAATTGTTGCTG |

**Supplementary Figure 1** RIP was performed using anti-TRIM21 and control IgG antibodies, followed by real-time PCR to examine the enrichment of lncTRPM2-AS. Bars, ±SD; n = 3, **P < 0.01.

**Supplementary Figure 2** The image of western blot shows that the domain was successfully constructed. WT full-length plasmid of TRPM2 (0-4662bp), Domain1 (0-1092bp), and Domain 2 (0-2937bp), all of which are tagged with Myc and co-transfected with lncTRPM2-AS in 293T cells. n = 3.

**Supplementary Figure 3** The TRPM2 channel activator cADPR was used to stimulate THP1 cells, and then flow cytometry was used to detect the changes in cellular ROS. n = 3.

**Supplementary Figure 4** The effects of two different calcium agonists on calcium in shAS cells. **A** After shNC and shAS cells were treated with TRPM2 activator cADPR, calcium was detected by flow cytometry. **B** ShNC and shAS cells were treated with voltage-gated calcium channel activator Bay-K-8644, and calcium was detected by flow cytometry. n = 3.
