# Supplementary figures and images for: LncTRPM2-AS inhibits TRIM21-mediated TRPM2 ubiquitination and prevents autophagy-induced apoptosis of macrophages in asthma
Source: Cell Death Dis. 2021 Dec 13;12(12):1153. doi: 10.1038/s41419-021-04437-6 (PMC8668916; doi:10.1038/s41419-021-04437-6)

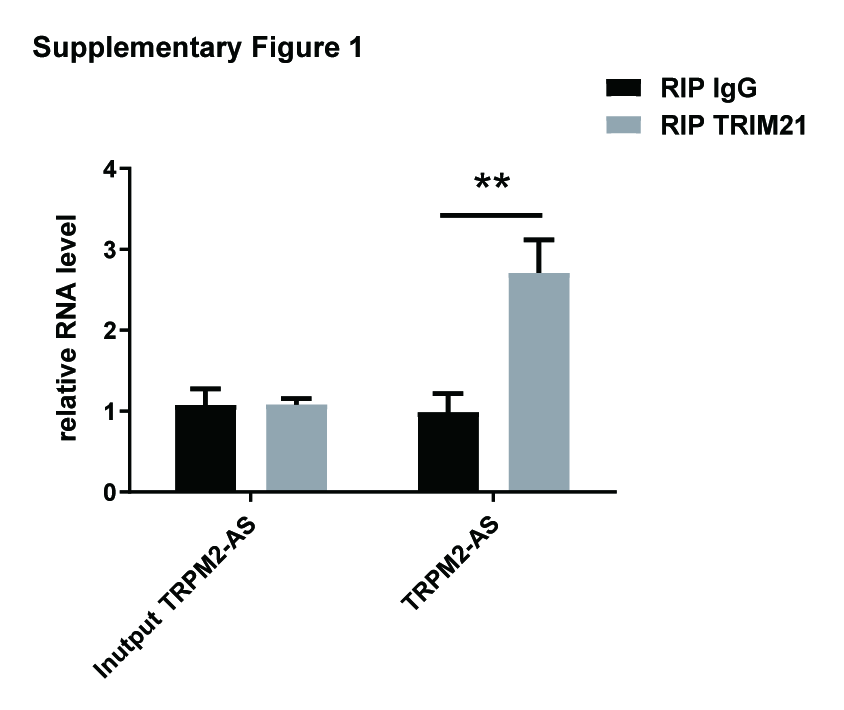

Supplement: Supplementary file 2 — Supplementary Figure 1 [file 41419_2021_4437_MOESM2_ESM.tif]

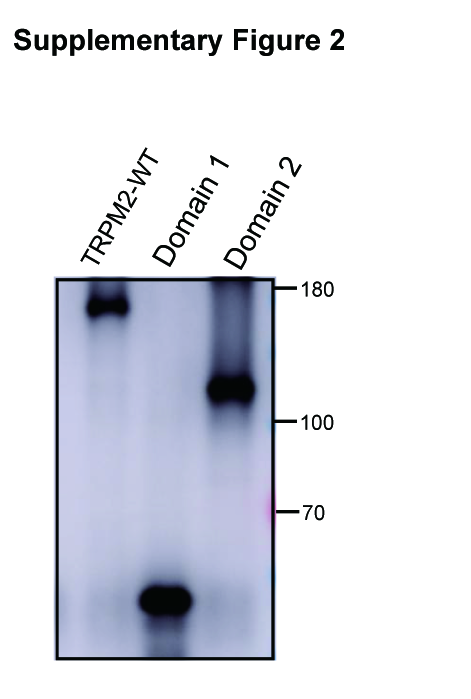

Supplement: Supplementary file 3 — Supplementary Figure 2 [file 41419_2021_4437_MOESM3_ESM.tif]

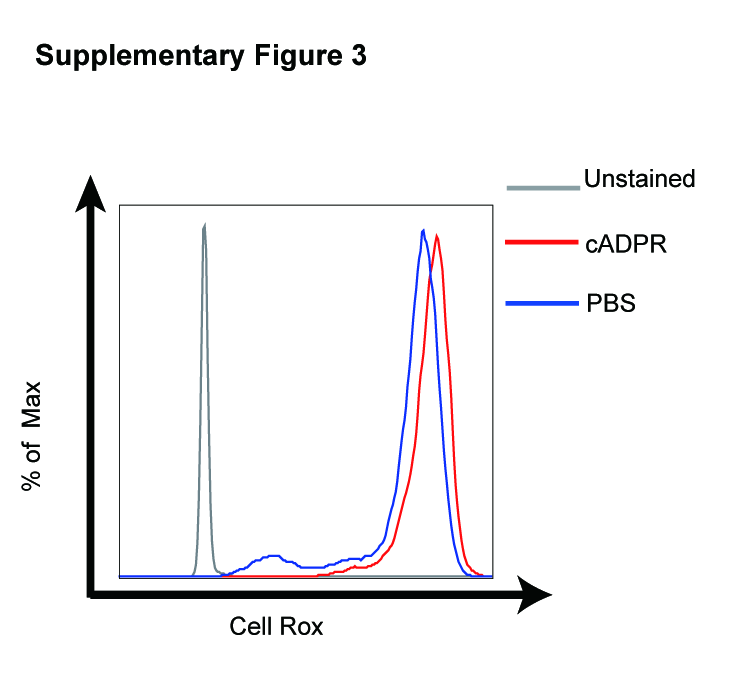

Supplement: Supplementary file 4 — Supplementary Figure 3 [file 41419_2021_4437_MOESM4_ESM.tif]

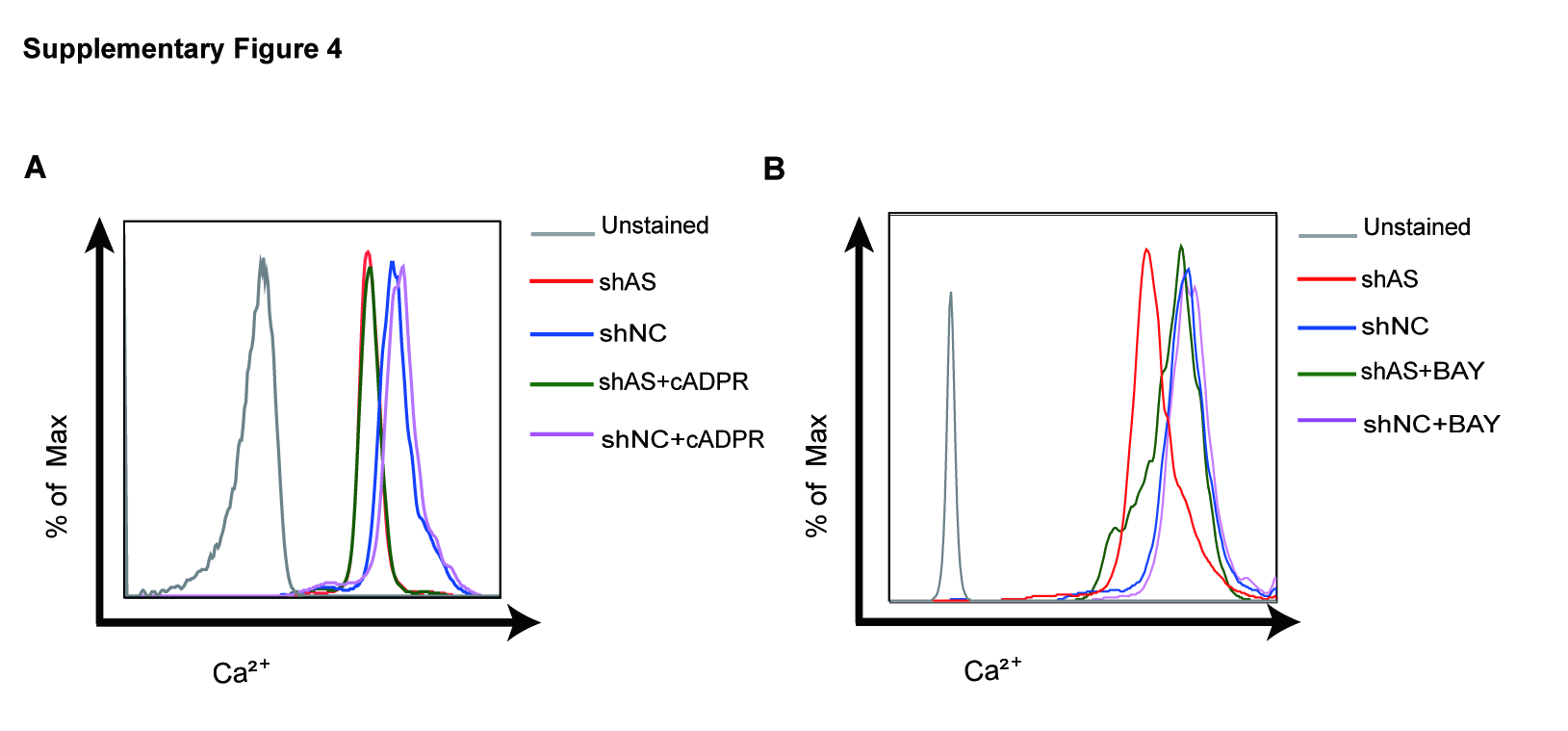

Supplement: Supplementary file 5 — Supplementary Figure 4 [file 41419_2021_4437_MOESM5_ESM.tif]
